# The American Mineral Springs and the Settlers

**Published:** 1917-02

**Authors:** Felix Von Oefele

**Affiliations:** New York


### The American Mineral Springs and the Settlers.

DR. FELIX VON OEFELE, New York.

The American mineral springs used by the settlers were originally mineral springs learned from the aboriginal Indians. Many Indian springs came into the use of the white man. Others have been lost and were later again discovered. But not all real mineral springs have been discovered by the Indians before the Caucasians arrived. The American areas have been too large and the density of Indian population was too slightly distributed over the entire continent for a complete control. The Kickapoo Magnetic Springs of Warren County, Indiana, were discovered by the Kickapoo Indians June, 1750. Restricted and pushed together into the reservations, the Indians got more opportunity to find a higher percentage of the local mineral springs. They were then too satisfied by a lower degree of therapeutic value in the new restrictions to call a spring a medicinal spring. Such springs were also taken some time by the settlers. Other mineral springs have never been used before by the aboriginal Indians.

The country of the modern United States had already been visited by the Normans from Greenland even about the year 1000. The next visits were in 1497 and in 1584 following the landing of Columbus. But the first settlers of modern United States started in 1606 in Virginia which in 1621 received a fairly liberal constitution. In 1612 New York was first settled. In 1620 the first settlers started in Massachusetts. The "New England Confederation" was formed in 1643 and New York became English in 1667. The Jesuits started to christianize California in 1690.

Virginia, New England including New York, and California became the three centers of practical American balneology and remained the principal districts of balneological interest.

Hunters and trappers used the old Indian mineral springs. We have no individual records; but we are able to abstract from imaginary stories of the literature. Colonel Davy Crockett after his famous single-handed fight with the Mexican lions in the vicinity bathed his wounds in Texas Sour Springs, a spring known to the Indians. Every settler tried to make a fortune in a different direction. The Caucasian settlers learned from the red men of the existence of remarkable mineral springs. Some of them expected to get rich obtaining the property of a powerful mineral spring.

About the end of the seventeenth century, the balneology of the old countries began a new development with the aid of chemistry. The Irishman, Robert Boyle, (1627-1691) detected the reaction of vegetable colors to alkalines and acids. The Frenchman Duclos adapted this method to a systematic research of the French mineral springs. Hierne, Valerius, Bouldue and many other chemists continued to render the balneological chemistry more perfect. The German physician Friedrich Hoffman (1660-1742), followed by Springsfeld and Venel paid particular attention to what they called the mineral spirit of waters and which has since been proved by Joseph Priestly (1733-1804) to be the carbonic acid gas. Carbonic acid at this time was called by different scholars very many different names; it is often termed fixed air. The Swedish chemist, Tobern Olof Bergman made a finishing stroke of perfection (1735-1784) by manufacturing artificial mineral waters. It was a remark of Bergman, that in all ages good physicians were desirous of establishing the salutary art on a firm foundation and have considered it incumbent on them, to submit to chemical analysis such waters as were famous for the cure of any disease.

The balneological analysts of these times resorted to qualitative reaction of color, to precipitation, to distillation, to obtain the proportions of their aerial contents, and to evaporation and to crystalization to find their fixed principles. The real results of these remote times were close to modern analytical determinations, but were obtained with more painstaking labor.

But America was disturbed by continuous wars between England, France and Spain during the first century of balneological chemistry. Since 1765 England had ruled the entire Northern America, Mexico excepted. The Revolution started in 1773. These circumstances retarded the scientific development of American Balneology.

For reconstructions of lost records of the early sixteenth century, the poem Hiawatha of Longfellow (1807-1882) is often used. Longfellow did not correctly separate coarse tribes, such as Ojibway, Iroquois and Dacota. The Dacota "midi"-water in the dialectic Teton shape of "minne" became very much misused on account of this poetry.

For the middle of the seventeenth century, Washington Irving's Sketch Book (1783-1859) shows truer local color. Rip Van Winkle gives some idea of the life of this time. It is useful to learn for detection of springs and other natural resources.

Shortly after 1690, a Mohawk chief treated a wounded French officer by one of the springs of Saratoga district. This indefinite story is the basis of priority claims of Saratoga Springs. Berkeley Springs, Virginia, claims an older settler history than Saratoga.

In the early part of the eighteenth century, Governor

Spottswood led an expedition from Williamsburg, the capital of the "Old Dominion" to the summit of the Blue Ridge. Not the least of the incentives of his followers was the hope of discovery of the wonderful health-restoring and live-giving waters of which so much had been told his people by the natives of the forests. He was not able to reach Virginia Hot Springs. Not many years elapsed before the sparkling thermal waters of the Hot Springs Valley were known and appreciated by the people of the whole colony.

In 1723, Beaver Lithia Springs were privileged.

In 1750, Stafford Springs, Tolland County, Connecticut went from Indian owners over to settlers.

Sir William Johnson, the English governor of New York, was the friend of the red man. He suffered from gout and was treated in 1767 by Indian medicine men with mineral springs of Saratoga County. He used Old Iron Springs of Ballston Spa at first. But what other springs he used, is an unsettled question; the answers are influenced by the later rivalry between Ballston Spa and Saratoga Springs. It was difficult to reach these springs. Sir William came in August 1767 to Schenectady and found an open road to McDonald's Clearing. A new way was opened through the forest and still has the name of the highway from Schenectady to Ballston Spa. The old spring corresponding to the Old Iron Spring was called "Public Well" or "Iron Railing Spring." There was a clearing there with residues of Indian camps. Later on Sir William used a spring situated further north. It is supposed to have been High Rock Spring. I do not agree that it was this spring. The people of the neighborhood used these springs therapeutically before Sir William; at least one of the McDonald brethren did. Sir William declared the Iron Spring of Ballston Spa public property. It remained the "Public Well" and was excepted as such in all the later contracts.

Sir William recovered in the fall of 1767 so far as to be able to walk the main part from Saratoga Springs to Schenectady on his own feet. This result started the fame of Saratoga Springs in high life.

In 1770, Dr. Constable of Schenectady reported medical effects from the Saratoga Springs.

In 1776, Asa Smith claimed the detection of Clarendon Springs, Vermont, and its medical usefulness.

In October 13, 1777, the English army was beaten near Saratoga, some miles east of Saratoga Springs. Some soldiers of the American army used the different mineral waters of Saratoga County partly as luxurious water, partly for medical indications. Dr. Samuel Tenney of Exeter was one of the physicians of this army and made observations and prescribed it.

In 1776, authentic records exist of patients actually treated at White Sulphur Springs, West Virginia, but the local tradition claims older use. The fame of this spring caused the adoption of the unscientific name of White Sulphur Springs or variations as Red Sulphur Springs, Black Sulphur Springs, etc., to many other American Springs.

In 1778, the Franciscan mission of Soledad received from the Mexican government Paraiso Hot Springs as private property in Monterey County, California.

In 1782, the first salt was produced from the springs of Mount Clemens, Michigan.

In 1783, George Washington, Governor George Clinton, Alexander Hamilton and Colonels Humphrey and Fish took a trip from Lake George to Schenectady. They went along Saratoga Springs and Ballston Spa. There were dense forests between both towns and twice Washington lost the right direction. The Old Iron Spring of Ballston Spa was furnished at this time only with a dug-in barrel. It was discovered in 1769 a short distance from Public Well.

On September 1, 1783, Samuel Tenney wrote a long letter to Dr. Joshua Fisher and gave in it an account of a number of Medicinal springs at Saratoga in the State of New York. He states the medical use of this water since about thirteen years before, at which time they were discovered by some surveyors. Dr. Young of Albany is known as a professional man prescribing the Saratoga water. The land for several miles round them was a wilderness. The springs have been considerably frequented by the poorer sort of people ever since their discovery. But for want of proper medical directions and necessary accommodations, their usefulness has been hithero much confined. These remarks concerned High Rock Spring and neighboring springs.

In 1784, Red Spring was discovered at Saratoga Springs.

In 1789, Gideon Putnam of Sutton went to Saratoga Springs and leased 300 acres of land. His ingeniousness first made Saratoga Springs a competitor of European mineral spring resorts.

Soon after the Revolutionary War, settlements were made in Hot Springs Valley of Virginia.

In the latter part of the eighteenth century, General George Rogers Clark made an expedition to Kaskaskia and Vincennes. He speaks in his memoirs of the West Baden Springs, Indiana, as a great resort.

In 1792, Congress Spring was discovered at Saratoga Springs.

In 1793, the above mentioned letter report of Samuel Tenney became published.

In 1793, Valentine Seaman (1770-1817) published a chemical analysis of a Saratoga mineral spring. He was promoted in 1792 at Philadelphia with a thesis on Opium and devoted himself with the fire of youth to the first chemical analysis of an American mineral spring water. He was furnished with all the respective analytical knowledges of his time. This publication forced competitors to similar analyses of their springs. The principal competitor was Ballston Spa.

In 1795, Dr. Vandervoort published the analysis of a Ballston water.

Maybe there are some older gas analyses. Seaman says: "No one has heretofore attempted to analyse these waters. All that has been done was a mere inquiry into the air discharged from them. See Dr. Mitchills experiments, related in the American Museum, vol. 4."

In 1800, the first therapeutic effect was seen with Poland Water, as tradition tells.

In 1802, "Old Springs" also called "Sweet Spring," the oldest spring of Salt Sulphur Springs, Monroe County, West Virginia, was discovered.

In 1802, Gideon Putnam built a part of Union Hall, on the place where Grand Union Hotel, Saratoga Springs, is now situated.

In 1803, Sans Souci Hotel was built at Ballston Spa in competition to Union Hall.

In 1804, the medicinal use of Bedford Springs at Bedford in Bedford County, Pennsylvania, started.

In 1805, many visitors of the new Bedford Springs were recorded.

In 1804, Hot Springs of Arkansas were discovered by Dunbar and Hunter in the district of the Quaquaw Indians.

In 1805, Salt Sulphur, the second spring of Salt Sulphur Springs, West Virginia, was discovered.

In 1805, Columbian Spring was discovered at Saratoga Springs.

In 1806, Hamilton Spring and Washington Spring were discovered at Saratoga Springs. History still shows the evidence of a strong competition of different individual spring owners within Saratoga Springs and with Ballston Spa. At this time Ballston Spa was victorious.

During the winter 1805-1806, Ballston Spa tried to be open as a winter resort.

In 1806, General Pike discovered Manitou Springs, Colorado, an old Indian spring resort.

In 1807, Arkansas Hot Springs starts its history as regular mineral spring place.

In 1808, there appeared in several of the American newspapers an analysis of Ballston Spa Water, said to have been made in France, but without the name of the analyst. It was reproduced in the Medical Repository of New York and again republished in the Monthly Anthology of Boston. This was a falsification.

In 1809, Valentine Seaman seemed to be influenced by Saratoga interests to reedit and augment the publication of 1793. He states the extraordinary accommodations and entertainment furnished at Ballston, equaling the most unbounded wish. He made an analysis of Ballston water, showing the impossibility of the claimed 300% volume of carbonic acid gas. He also made an analysis of the tepid akratotherm Lebanon Springs, stating some experiments done there by the Professors Mitchill, Waterhouse and Post. He recognized the gas of this spring as Nitrogen. He also mentioned four miles west of High Rock Spring a "strong scented sulphureous spring." He mentioned Bedford, Pennsylvania, also as a famous well known mineral spring.

In 1811, the mineral spring of Middletown Mineral Springs, Rutland County, Vermont, used by the Indians since time immemorial was first therapeutically used by the settlers. It contains about 0:017% total solids.

In 1818, the district of Arkansas Hot Springs went from the Quaquaw Indians to the Federal government.

In 1821, Indian Springs, Georgia., with 1000 acres land was excepted from a cession of the Creek nation.

Before the year 1814, Indian Springs, Martin County, Indiana, first used by settlers.

In 1821, Iodine Spring was discovered at Salt Sulphur Springs, West Virginia.

In 1825, Dr. Church of Pittsburgh analyzed the Bedford Magnesia Water.

In 1826, Chase built the bathing house at St. Catharine's Well. This spring may have been used in the years before.

In 1830, Dearborn Spring, Vermont, was discovered and rapidly became famous in the neighborhood.

In 1832, the site of the present springs property of West Baden, Indiana, was first bought from the government.

In 1834, a company of Maryland and Virginia men was formed to develop Fauquier White Sulphur Springs, Virginia. Two large hotels and a number of cottages capable of accommodating 1000 persons were built.

In 1834, the real spring district of Arkansas Hot Springs was declared a Federal reservation.

In 1835, Putnam Spring and Star Spring were discovered at Saratoga Springs.

In 1836, G. Froost made an incomplete analysis of Ferndale Springs, Tennessee.

In 1839, Pavilion Spring and United States Spring were discovered at Saratoga Springs.

In 1840, West Baden, Indiana, were developed into a health resort. Dr. John A. Lane built the first hotel.

In 1846, Empire Spring was discovered at Saratoga Springs.

In 1847, Grand View Sanatorium, Berks County, Pennsylvania, was built; it contained Pavilion Spring.

In 1848, Moorman Mineral Well, Washtenaw, County, Michigan, became used.

In 1849, the legislature of Virginia held a summer session at Fauquier White Sulphur Springs, Virginia.

In 1849, the old fashionable Sans Souci Hotel at Ballston Spa was transformed into a State and National Law School. Saratoga Springs was now evidently more successful and the fame of Ballston Spa was broken down.

In 1850, Agua Caliente, Arizona, became the property of King Wolsey. It was within the district of the Apaches. Three times he was plundered by the Indians in vengeance for taking the spring. The ladies bathed in the spring water guarded by armed sentinels on the neighboring hills. Brush shacks were used instead of bath houses.

In 1850, Henry Foster, M. D., built the sanitarium at Clifton Springs. The springs were known before and used by the inhabitants.

About 1850, Powder Springs, Cobb County, Georgia, were discovered.

In 1851, Sans Souci Hotel at Ballston Spa tried again to be a hotel.

In 1855, Dr. Strong's "The Saratoga Spring Sanitarium" was built.

In 1859, Excelsior Spring was discovered at Saratoga Springs.

In 1859, the first professional medical paper concerning Poland Springs, Androscoggin County, Maine, was published.

In 1861, the spring resorts of the southern Appalachian Mountains were well equipped and frequented by wealthy visitors, but they lost all by the civil war, for instance Gailbroth Springs, Tennessee; Greenbrier White Sulphur Springs, West Virginia.

In 1862, the two hotels of Fauqier White Sulphur Springs were burned during a battle.

In 1863, Sans Souci Hotel at Ballston Spa became a boarding school for girls.

In 1865, the first well was drilled at Mount Clemens, Michigan, prospecting for oil, brine was found.

In 1865, Saratoga A Spring and Seltzer Spring were discovered at Saratoga Springs.

In 1865, Cloverdale Lithia Springs, Cumberland County, Pennsylvania, were found as an artesian well by searchers of oil.

After this time Equinox Springs was very famous and was much visited by people from entire New England.

Since the later years of this decade, Professor Chandler made many mineral spring analyses, which are exact and still very useful.

In 1868, Union Spring, Hathorn Spring and Eureka Spring were discovered at Saratoga Springs.

In 1869 and 1870, Eaton Rapids Wells, Eaton County, Michigan, was discovered.

In 1871, the sale of water of Deep Rock Spring, Oswego County, New York, started.

In 1872, Saratoga Vichy, N. Y., was drilled.

In 1873, the first regular bath house was completed at Mount Clemens, Michigan; the brine was used for therapeutical purpose.

In 1876, Fort Crawford Mineral Well was discovered.

In 1876, the Poland Spring House was built for regular cures.

In 1877, a company was formed for the purpose of restoring Fauquier White Sulphur Springs, Virginia; 500 accommodation to 1000 few decades ago.

In 1878, Prof. F. A. Genth again analyzed Bedford Magnesia Springs.

In 1879, Plainfield Sanitarium, New Jersey, was built, claiming a sulphur spring (?).

Up to 1880, Glenwood Springs, Colorado, was included in the Ute Reservation and was not accessible for White Visitors.

In 1881, Man-a-cea Spring was found near Independence, Preston County, West Virginia. The summer was very dry. The vicinity of the spring kept green grass. It was originally used for domestic purposes. The business started later. The name is evidently coined for the purpose of imitating old Indian names and may have first been Panacea.

---

**Lamblia Infection in England.** A. Malins Smith and J. R. Matthews, B. M. J., Sept. 16, report three cases apparently autochthonous, only one of the men having been away from England and he for only two days in Holland. (Mac Farland mentions lamblia only in a foot note as a probably harmless parasite and makes no statement as to its distribution).

